# miR455 is linked to hypoxia signaling and is deregulated in preeclampsia

**DOI:** 10.1038/cddis.2014.368

**Published:** 2014-09-04

**Authors:** S Lalevée, O Lapaire, M Bühler

**Affiliations:** 1 Laboratory for Prenatal Medicine, Department of Biomedicine, University Hospital Basel, Basel CH-4031, Switzerland; 2 Department of Obstetrics and Gynecology, University Hospital Basel, CH-4031 Basel, Switzerland; 3 Friedrich Miescher Institute for Biomedical Research, Maulbeerstrasse 66, CH-4058 Basel, Switzerland; 4 University of Basel, Petersplatz 10, CH-4003 Basel, Switzerland

## Abstract

Preeclampsia is a severe pregnancy-related disorder and a leading cause of maternal and fetal mortality worldwide. Early identification of patients with an increased risk for preeclampsia is thus one of the most important goals in obstetrics. Here we identify two related human microRNAs as potential biomarkers to detect at-risk pregnancies. We demonstrate that miR455-3P and miR455-5P are significantly downregulated in placentas from preeclampsia patients, whereas other placenta-specific microRNAs remain unaffected. microRNA target prediction and validation revealed a potential link of miR455-3P to hypoxia signaling. Together with our observation that expression levels of miR455-3P and miR455-5P are upregulated during trophoblast differentiation, our results suggest a model in which miR455-3P represses a hypoxia response that might otherwise prevent cytotrophoblasts from syncytiotrophoblast differentiation. In summary, our work reveals aberrant hypoxia signaling in preeclampsia that can be explained by deregulated expression of miR455. As miR455 has been found in circulating blood, the development of noninvasive prenatal tests enabling early diagnosis of preeclampsia may be possible.

The placenta connects the developing fetus to the uterine wall and allows gas exchange, nutrient uptake, and elimination of waste products via the mother's blood supply. Moreover, the placenta has endocrine activity, producing various pregnancy-associated hormones and growth factors that regulate fetal growth and the maternal response to the pregnancy.^1^ Aberrant function or development of the placenta has been associated with many pregnancy complications, including preeclampsia (PE). PE is a multisystemic, pregnancy-associated disorder with an incidence of 2–5% that is a major cause of maternal and fetal morbidity and mortality.^2^ Although the exact etiology of PE remains elusive, the placenta has a central role.^3^ In the first and second trimester, local aberrant feto-maternal immune interactions within the uterine wall lead to impaired arterial wall invasion by trophoblast cells. This results in failed transformation of the uterine spiral arteries and subsequently decreased placental perfusion.^3^ Chronic hypoxia or alternate periods of hypoxia/reoxygenation within the intervillous space trigger tissue oxidative stress and increase placental apoptosis and necrosis.^4^ Subsequently, placental debris and the aberrant expression of pro-inflammatory, antiangiogenic and angiogenic factors lead to systemic endothelial cell dysfunction and an exaggerated inflammatory response.^5, 6, 7, 8, 9^ Interestingly, the particles shedding at the surface of the placenta are released into the maternal circulation and their content, DNA as well as microRNAs (miRNAs) , may serve as noninvasive biomarkers for pregnancy-related disorders.^10, 11^

MicroRNAs are a large family of post-transcriptional regulators of gene expression, circa 21 nucleotides (nt) in length, that control many developmental and cellular processes in eukaryotic organisms. MicroRNAs are processed from precursor molecules (pri-miRNAs), which are either transcribed from independent miRNA genes or represent introns of protein-coding genes. Pri-microRNAs fold into hairpins that are sequentially processed by the nuclear RNAse III enzyme Drosha into pre-miRNAs of ∼70 nt. After export to the cytoplasm, the pre-miRNA is further processed by Dicer to a 21-bp miRNA/miRNA* duplex. One strand of this duplex, representing a mature miRNA, is then incorporated into the miRNA-induced silencing complex (miRISC).^12^ For many miRNA genes, one mature miRNA derived from the 5′ or the 3′ arm of the pre-miRNA hairpin is preferentially incorporated into miRISC. However, around 10–15% of miRNA genes express both mature miRNAs. These are annotated using -5p and -3p suffixes.^13, 14, 15^

As part of miRISC, mature miRNAs base pair with sequences in the 3′-UTR of target mRNAs and direct their translational repression and/or mRNA deadenylation and degradation.^12^ MicroRNAs have also been suggested to target the coding sequence of some mRNAs as well as the 5′UTR of ribosomal protein-coding mRNAs, leading to inhibition or activation of the targets, respectively.^16, 17^ Most animal miRNAs imperfectly base pair with target mRNAs. Nevertheless, efficient mRNA targeting requires continuous base pairing of the miRNA ‘seed' sequence (nt 2–8).^18^ Because complementarity more extensive than seed pairing is unusual in animals, predicting miRNA target mRNAs computationally has remained a challenge. Nonetheless, several computational tools for predicting potential miRNA targets have been developed.^18^

Profiling of miRNA expression has revealed that some miRNAs are expressed universally but others tissue specifically.^19^ Accumulating evidence shows that miRNAs are frequently deregulated in human malignancies and can act as oncogenes or tumor-suppressor genes.^20, 21^ In the human placenta, two large clusters of miRNA genes are encoded on chromosome 14 (C14MC) and chromosome 19 (C19MC).^22, 23^ Interestingly, expression of certain placenta-specific miRNAs is deregulated in cancer tissues, although their functional roles have remained elusive.^23, 24^ Few placental-specific miRNAs have been associated with placental disorders such as PE.^25^ For example, several studies have revealed upregulation of the miRNA miR210 in placenta from PE patients.^26, 27, 28, 29, 30^ However, most of these studies were limited by the scarcity of placental samples needed for miRNA expression, their heterogeneity, and/or the low number of miRNAs studied.^26, 30^ Thus, it is not clear to what extent miRNAs other than miR210 are differentially expressed in PE patients.

Trophoblasts are specialized cells of the placenta that have an important role in embryo implantation and interaction with the maternal uterus. Two different trophoblast differentiation pathways lead to placental development.^31^ In the extravillous pathway, cells differentiate either into interstitial extravillous trophoblasts that invade the decidua and a part of the myometrium, or into endovascular extravillous trophoblasts that remodel the maternal vessels. In the villous pathway, cytotrophoblast (CT) cells fuse to a multinucleated syncytiotrophoblast (SCT) layer that covers the entire surface of the placenta.^31^ This syncytium is in direct contact with maternal blood and thus facilitates the exchange of nutrients, wastes, and gases between the maternal and fetal systems. Defective CT to SCT differentiation has been proposed to be involved in the etiology of PE.^32^

To study miRNA expression during villous trophoblast differentiation, we employed an established *in vitro* model that recapitulates the differentiation of CT cells into SCT and profiled miRNA expression by next-generation small RNA sequencing. This analysis revealed two related miRNAs (miR455-5P/-3P) that were reproducibly upregulated upon CT to SCT differentiation. The results of target prediction and validation analyses suggest that miR455-3P restrains a hypoxia response that would otherwise prevent CT to SCT differentiation. Importantly, we found that expression of miR455 was significantly downregulated in 15 PE cases compared with 14 healthy donor controls, whereas the levels of other placenta-specific miRNAs remained unaffected. Therefore, miR455 miRNAs are potential biomarkers for early diagnosis of at-risk pregnancies.

## Results

### *In vitro* reconstitution of cytotrophoblast to syncytiotrophoblast differentiation

**Figure 1:**
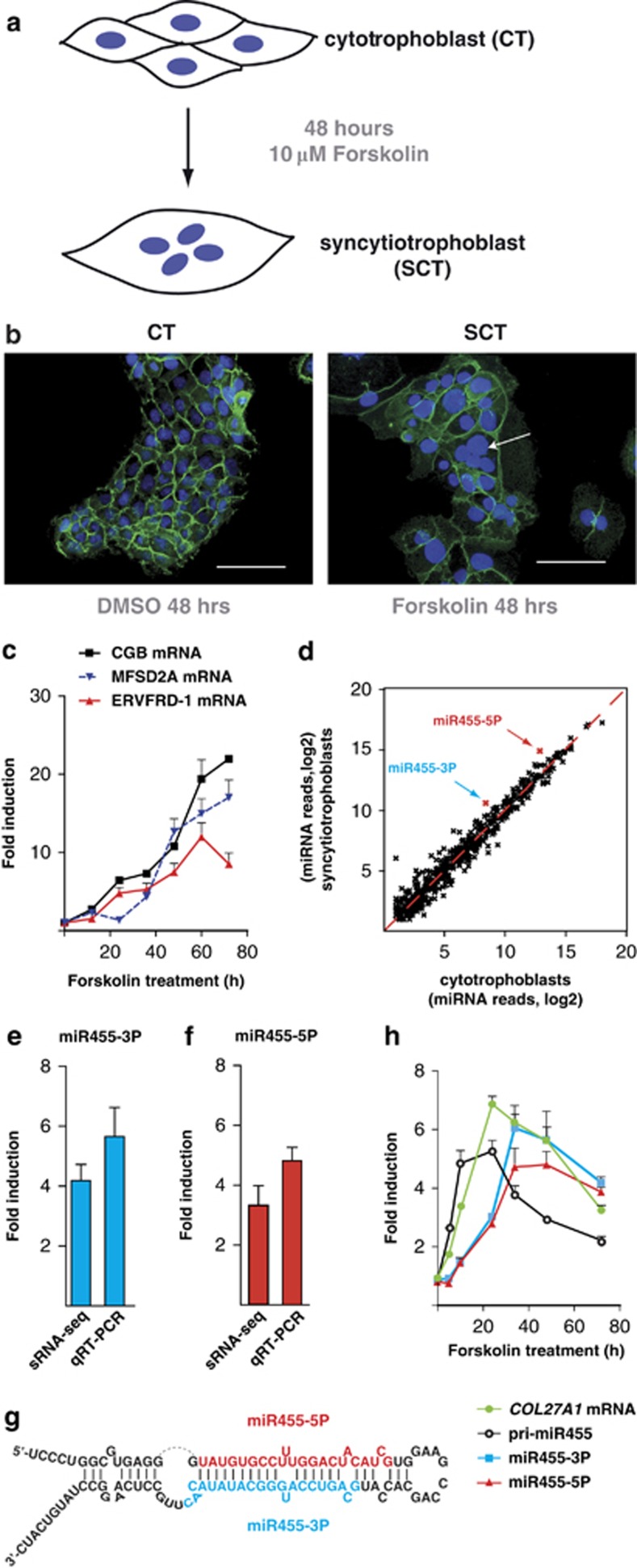
miR455 is induced upon *in vitro* syncytialization. (**a**) Schematic diagram of cyto- (CT) to syncytiotrophoblast (SCT) differentiation. (**b**) Fusion of BeWo cells following FSK treatment. Cells were fixed and immunostained using anti-E-cadherin antibody (green). Nuclei were counterstained with DAPI (blue). Left panel: BeWo cells were treated with vehicle (DMSO) for 48 h and remained as mononucleated (CT) cells. Right panel: cells started to fuse after 48 h of FSK treatment, producing multinucleated (SCT) cells (white arrow). Scale bar, 100 *μ*m (**c**) Expression of SCT markers. RNA was extracted at different time points after FSK treatment. mRNA levels of three SCT-specific genes were measured by qRT-PCR. Data were normalized to RPLP0 mRNA and compared to control treatments (median value ± S.E.M. of three independent experiments). (**d**) miR455 induction in SCT *versus* CT. After 48 h of control or FSK treatment, total RNA was extracted and small RNAs subjected to high-throughput sequencing. Normalized miRNA levels of control- and FSK-treated cells are plotted as a log_2_ scale on the x and y axes, respectively. Each miRNA is represented by a cross. Deregulated miR455 miRNAs are indicated by red crosses. The result of one representative biological replicate is shown. (**e** and **f**) Fold induction of miR455-3P (E) and -5P (F) after 48 h of FSK treatment was determined by small RNA sequencing or qRT-PCR (median value ± S.E.M. of four and three independent experiments, respectively). (**g**) Hairpin structure of the pre-miR455 precursor transcript. (**h**) Expression analysis of pri-miR455, miR455, and of the host gene *COL27A1*. RNA was extracted at the indicated time points after FSK treatment and analyzed by qRT-PCR using TaqMan assays. Data were normalized to U6 snRNA or RPLP0 mRNA and compared to control treatments (median value ± S.E.M. of three independent experiments)

Because the placenta is a complex and heterogeneous organ, detailed molecular study of the mechanisms underlying placental biology is very challenging, if not impossible. Therefore, the use of appropriate cellular models is advantageous. To study miRNAs during villous trophoblast cell differentiation, we exploited the established CT-like cell line (BeWo). BeWo cells have been shown to syncytialize upon treatment with forskolin (FSK), an adenylate cyclase activator and a cyclic AMP inducer (Figure 1a).^33^ Indeed, staining control-treated cells with DAPI together with an antibody recognizing the plasma membrane marker E-cadherin confirmed that BeWo cells are mononucleated. However, treatment with 10 *μ*M FSK promoted the formation of multinucleated cells, thus demonstrating SCT formation (Figure 1b).

To further confirm CT to SCT transition upon FSK treatment, we monitored the expression of genes induced during syncytialization using quantitative RT-PCR. Beta chorionic gonadotropin hormone (CGB) is a marker of SCT formation.^34^ FSK treatment produced a gradual increase in CGB mRNA levels to a maximum after 60 h. Similarly, expression of ERVFRD-1 and MFSD2A was strongly induced upon FSK treatment (Figure 1c). ERVFRD-1 is an endogenous retroviral gene that encodes for the syncytin2 protein, and MFSD2A encodes for the syncytin2 receptor. Both proteins are essential for syncytialization.^33, 35^ These results confirm effective induction of syncytialization upon FSK treatment and validate BeWo cells as a suitable model to study CT to SCT differentiation.

### miR455 is differentially expressed during syncytialization

To investigate whether the syncytialization process is accompanied by changes in miRNA expression, we isolated small RNAs from four independent *in vitro* differentiation experiments and generated libraries for Illumina sequencing. After processing the sequencing data and filtering for miRNAs annotated in miRBase (http://www.mirbase.org/), we compared miRNA expression profiles of the different biological replicates. MicroRNA expression was highly correlated between all four biological replicates, for both control- and FSK-treated cells (Supplementary Figure 1). Confirming the trophoblastic origin of the BeWo cell line, we found that 50% of all miRNAs sequenced from either control- or FSK-treated cells were derived from the chromosome-19-miRNA-cluster (C19MC) (Supplementary Table S1). C19MC encodes for 59 miRNAs that are expressed mainly in human placenta.^23^ Comparison of the expression of C19MC in FSK- *versus* control-treated cells showed no significant difference in expression of these placenta-specific miRNAs (Supplementary Table S1). Furthermore, overall miRNA expression profiles were remarkably similar in FSK-treated and control samples (Figure 1d and Supplementary Figure 1). However, elevated levels of the miRNAs miR455-3P and miR455-5P were observed consistently in FSK-treated cells. To validate this observation, we assessed miR455-3P and miR455-5P expression by qRT-PCR using validated TaqMan assays. Consistent with the small RNA sequencing data, miR455-3P and miR455-5P levels were enhanced ca. fivefold when cells were treated with FSK for 48 h (Figures 1e and f).

The mature miR455-3P and miR455-5P miRNAs both derive from a pre-miRNA hairpin encoded in intron 10 of the collagen gene *COL27A1* (Figure 1g and Supplementary Figure 2). Thus, the elevated levels of the two mature miRNAs may be the result of increased transcription of the *COL27A1* host gene. Consistent with this, we observed a strong increase in *COL27A1* mRNA levels upon FSK treatment of BeWo cells. *COL27A1* mRNA levels were highest 24 h after FSK treatment and declined gradually thereafter (Figure 1h). Importantly, we observed a very similar expression profile for the pri-miR455 precursor transcript upon FSK treatment, though not as high as the maximal level of the *COL27A1* mRNA (Figure 1h). Furthermore, increase in mature miR455-3P and miR455-5P miRNAs occurred later than the pri-miR455 precursor transcript. We observed a maximal increase in mature miR455 ca. 48 h after FSK treatment, concomitant with a decline in pri-miR455 (Figure 1h). These results demonstrate that treatment of BeWo cells with FSK stimulates the expression of the *COL27A1* gene and thus leads to increased production of pri-miR455. Therefore, although increased precursor processing or miRNA stability cannot be ruled out, the observed elevation in mature miR455 upon FSK treatment of BeWo cells can be attributed to increased expression of the *COL27A1* gene.

### Deregulation of miR455 in placentas from PE patients

**Figure 2:**
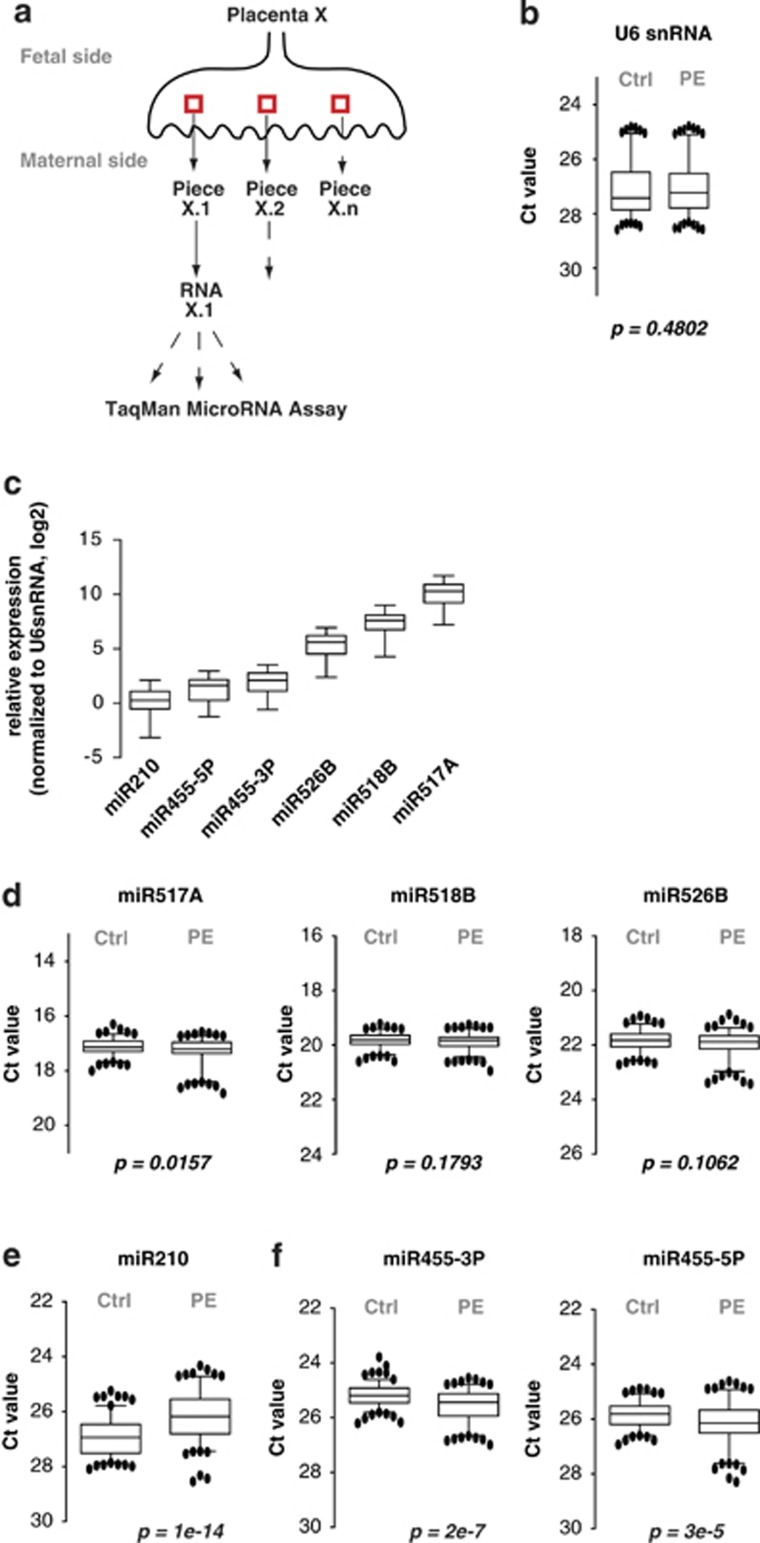
miR455 and miR210 are deregulated in preeclampsia (PE) placentas. (**a**) Schematic diagram of placenta processing. Circa 3–4 pieces (red box) were dissected from the inner part of each placenta (X) to limit maternal contamination. Total RNA was extracted from each placenta piece (X.1 to X.n) and miRNA expression levels measured in three technical replicates by qRT-PCR using TaqMan assays. (**b**) U6 snRNA expression in PE and control (Ctrl) placentas. U6 snRNA levels were measured by TaqMan qRT-PCR. The cycle threshold (Ct) value obtained for U6 snRNA is plotted as a whiskers box plot 5th–95th percentile representation. The *P*-value was calculated by Mann–Whitney test. (**c**) miRNAs expressed in placentas. The expression of six selected miRNAs was determined by TaqMan qRT-PCR in control placentas. For each miRNA, expression was normalized to U6 snRNA and plotted on a log2 scale using a whiskers box plot 5th–95th percentile representation. (**d**) MicroRNAs that are not expressed differentially in control *versus* PE placentas. (**e**) miR-210 levels are higher in PE than control placentas. (**f**) miR-455 levels are lower in PE than control placentas. (**d–f**): *P*-values were calculated by Mann–Whitney test

It has been proposed that PE involves irregular CT to SCT differentiation.^32^ To investigate whether miR455 is expressed in placenta and potentially misregulated in PE, we collected placenta samples from 15 PE cases and 14 healthy donor controls. The main clinical characteristics of the patients are summarized in Table 1. Notably, maternal age, body mass index, and percentage of nulliparity were not significantly different between the two patient groups. However, PE patients showed a tendency for intrauterine growth retardation, increased blood pressure (systolic and diastolic), and proteinuria, and gave birth on average 4 weeks earlier than the control group. Placentas were dissected from the villus tree immediately after the delivery. To compensate for intra-placental variability, we extracted total RNA from 3–4 independent samples per placenta (Figure 2a), giving totals of at least 45 PE and 42 control RNA samples.

qRT-PCR analysis revealed that U6 small nuclear RNA (snRNA) expression levels were not significantly different between the two patient groups (Figure 2b). Further validating the quality of our samples, we detected miR526B, miR518B, and miR517A, three representative miRNAs encoded in the placenta-specific C19MC, as well as miR210, an miRNA shown previously to be upregulated in placenta from PE patients^26, 27, 28^ (Figure 2c). Importantly, we also detected miR455-3P and miR455-5P, which were both more abundant than U6 snRNA and miR210 in the control RNA samples (Figure 2c).

Comparison of miRNA abundance in samples from the two patient groups showed no significant differences in miR526B, miR518B, or miR517A, demonstrating that expression of the C19MC was not notably affected in PE patients (Figure 2d). However, miR210 was significantly upregulated in placenta from PE patients (Figure 2e), consistent with previous reports.^26, 27, 28, 29, 30, 36, 37^ In contrast, miR455-3P and miR455-5P levels were significantly lower in PE than in control samples (Figure 2f).

In summary, the mature miRNAs miR455-3P and miR455-5P are expressed in human placenta. Whereas expression of miRNAs from the placenta-specific C19MC was not affected, miR455 miRNA levels were significantly lower in placenta from PE than control patients. On the contrary, miR210 was more abundant in PE samples.

### miR455 miRNAs are part of functional miRISC

**Figure 3:**
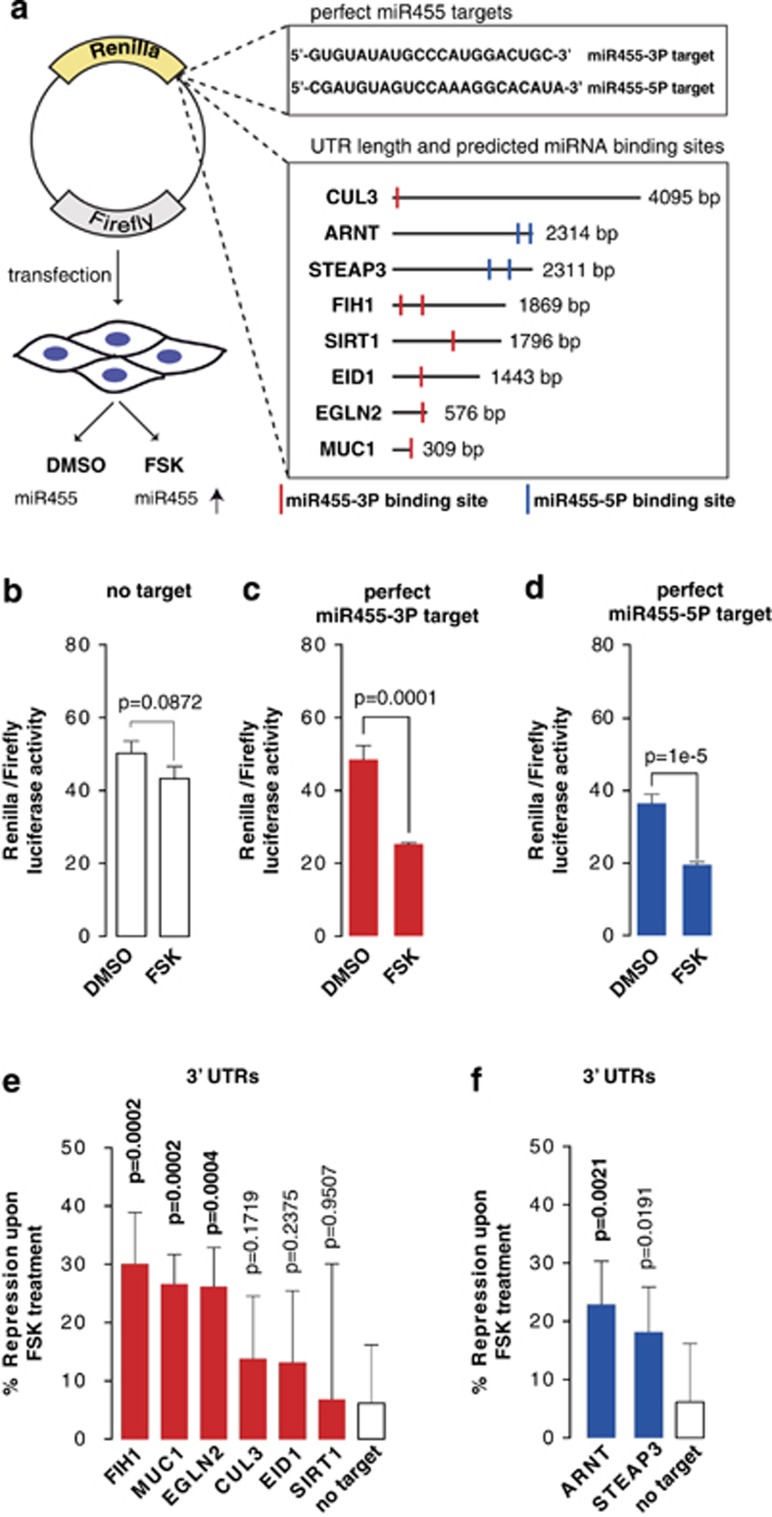
miR455 is part of functional miRISC in BeWo cells. (**a**) Schematic of the dual luciferase assay used in this study. The exact complementary sequences of miR455-3 P and -5P, or the 3′UTRs of eight potential target genes of miR455, were cloned 3′ to the renilla luciferase (RL) ORF (upper lanes). Positions of the potential binding sites of miR455-3P (red) or miR455-5P (blue) are indicated. BeWo cells were transfected and treated for 48 h with either FSK (high miR455 levels) or DMSO (low miR455 levels). (**b**) FSK has no effect on renilla luciferase expression. Vector with no target sequences cloned 3' of renilla was transfected into BeWo cells. After 48 h of treatment, cells were lysed and the RL and FL activities were measured. RL/FL ratios were calculated for DMSO- and FSK-treated cells. *P*-values were calculated by the non-parametric *T*-test. (**c** and **d**) Vectors with miR455-3P or -5P target sequences cloned 3' of renilla were transfected into BeWo cells. RL/FL ratios were calculated for DMSO- and FSK-treated cells. *P*-values were calculated by the non-parametric *T*-test. (**e** and **f**) The 3′UTRs of eight potential miR455 targets were cloned into the dual luciferase plasmid. One day after transfection, cells were treated for 48 h with FSK or DMSO. RL/FL ratios were calculated for DMSO- and FSK-treated cells. Percentage repression of the respective miRNA target reporters was calculated by normalizing the RL/FL ratios after FSK treatment to the RL/FL rations after control treatment. *P*-values were calculated by the non-parametric *T*-test

As part of miRISC, mature miRNAs base pair to target mRNAs and direct their repression. Because miR455-3P and miR455-5P are relatively abundant in both BeWo cells and placenta samples, they might have an important role in regulating pathways relevant to placenta physiology. To test whether miR455-3P and miR455-5P are part of functional miRISC and to verify predicted miR455 targets, we adopted a dual luciferase-based miRNA-activity reporter assay.^38^ This assay comprises a mammalian expression vector encoding renilla luciferase (RL) and firefly luciferase (FL) reporter genes. The RL reporter gene is fused to a putative miRNA target sequence and thus monitors miRNA activity. The FL reporter gene is used as a normalization control (Figure 3a).

We first transfected BeWo cells with a reporter plasmid lacking miRNA target sequences. Following transfection, the cells were treated with either DMSO or FSK to induce syncytialization. We observed RL/FL activity ratios that were not significantly different in control- and FSK-treated cells, demonstrating that luciferase activity is not affected by the syncytialization process or by FSK treatment *per se*, in the absence of an miR455 target sequence (Figure 3b). However, RL activity was significantly reduced by FSK treatment when the RL reporter was fused to a fully complementary miR455-3P target sequence (Figure 3c). Similarly, RL fused to a complementary miR455-5P target sequence was repressed upon FSK treatment (Figure 3d). These results demonstrate that both miR455 miRNAs are part of functional miRISC in BeWo cells and that the miRNA activity assay reliably reports miR455-RISC activity.

### MUC1 mRNA is a physiological target of miR455-3P

To identify potential miR455 target mRNAs, we employed the miRNA target prediction software miRecords.^39^ Compiling lists of potential targets for miR455-3P and -5P, we noted at least eight genes that have been linked to hypoxia signaling (Figure 3a). This raised our interest because miR210 expression is stimulated by hypoxia-inducible factors (HIF),^40, 41, 42^ suggesting a potential hypoxia-related relationship between the three miRNAs we found to be differentially expressed in PE samples.

To validate the predicted miR455-3P and -5P target mRNAs, we first performed dual luciferase miRNA reporter assays in BeWo cells. We fused the complete 3′UTRs of the predicted mRNAs to the RL reporter and tested miR455-mediated repression in FSK *versus* control conditions (Figure 3a). In contrast to the RL reporter fused to a fully complementary miR455 binding site (Figures 3c and d), FSK treatment did not result in significant repression of RL when fused to the 3′ UTR of either CUL3, EID1, SIRT1, or STEAP3 (Figures 3e and f). However, the 3′UTRs of EGLN2, MUC1, FIH1, or ARNT did produce significant RL repression upon FSK treatment (Figures 3e and f). These results validate the predicted miR455-5P binding sites in the 3′UTR of ARNT and the miR455-3P binding sites in the 3′UTR of EGLN2, MUC1, and FIH1.

**Figure 4:**
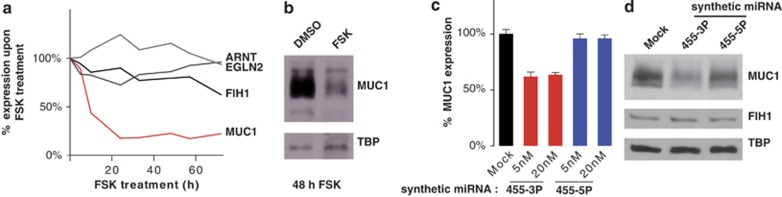
MUC1 is a *bona fide* target of miR455-3P in BeWo cells. (**a**) Expression of potential miR455 target mRNAs in FSK-treated BeWo cells. Expression of the indicated potential miR455 target mRNAs was analyzed by qRT-PCR. Values were normalized to RPLP0 and displayed relative to DMSO-treated cells. (**b**) MUC1 protein levels are reduced by FSK treatment. MUC1 and TBP protein levels were analyzed by western blotting 48 h after treatment. (**c** and **d**) MUC1 is repressed by miR455-3P but not miR455-5P. BeWo cells were transfected with synthetic miRNAs at two concentrations (5 and 20 nM). RNA and protein samples were harvested 48 h post-transfection. MUC1 mRNA and protein levels were determined by qRT-PCR (**c**) and western blotting (**d**), respectively. One western blot representative of a transfection with 20 nM synthetic miRNA is presented in **d**. TBP served as a loading control

To test whether the endogenous EGLN2, MUC1, FIH1, and ARNT mRNAs are under negative control by the miR455 miRNAs, we assessed mRNA and protein levels by qRT-PCR and western blotting, respectively. For EGLN2 and ARNT, neither mRNA nor protein levels changed significantly upon FSK treatment of BeWo cells (Figure 4a and Supplementary Figure 3). FIH1 was consistently only slightly repressed (Figure 4a and Supplementary Figure 3). In contrast, MUC1 was strongly repressed at both the mRNA and protein levels (Figures 4a and b and Supplementary Figure 3). To confirm miR455-3P-mediated *MUC1* mRNA repression independently of FSK treatment, we transfected BeWo cells with synthetic miR455 miRNAs. As expected, *MUC1* mRNA and protein levels were reduced by transfection of synthetic miR455-3P but not miR455-5P (Figures 3c and d). Thus, we conclude that *MUC1* mRNA is a *bona fide* miR455-3P target that is strongly repressed during FSK-induced syncytialization of BeWo cells.

### miR455-3P constrains HIF2A-mediated hypoxia signaling

**Figure 5:**
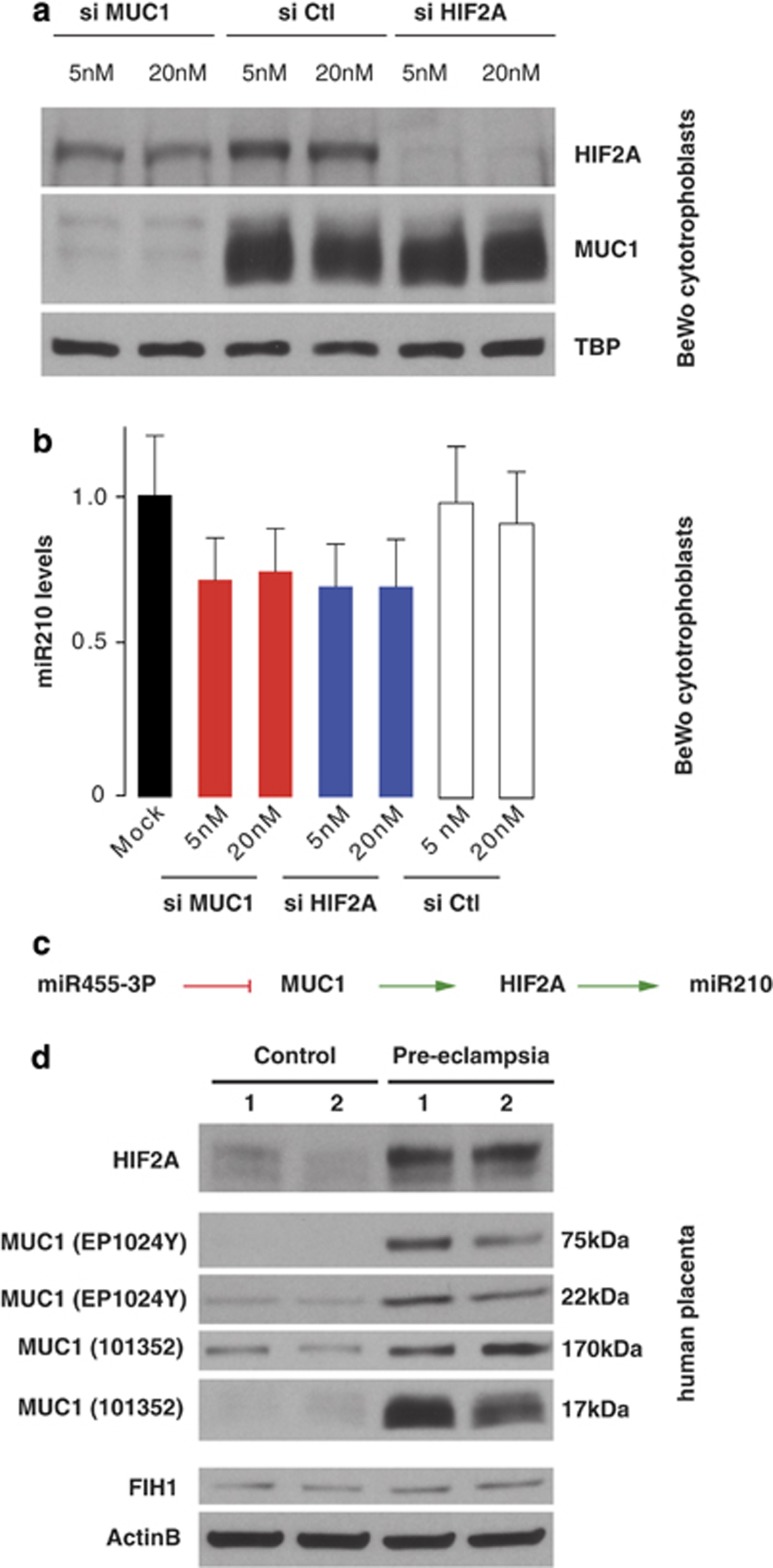
MUC1 and HIF2A are deregulated in PE placentas. (**a**) HIF2A is positively regulated by MUC1. BeWo cells were transfected with siRNAs against HIF2A or *MUC1* mRNA at two concentrations (5 and 20 nM) and with an All Star Negative Control siRNA. At 48 h posttransfection, cells were harvested and protein samples analyzed by western blotting. (**b**) miR210 is regulated by HIF2A and MUC1. Cells were harvested 48 h posttransfection and miR210 expression analyzed by TaqMan assays. Values were normalized to U6 snRNA expression (median value ± S.E.M. of three independent experiments). (**c**) Schematic representation of the miR455-3 P/miR210 signaling pathway. (**d**) MUC1 and HIF2A protein levels are upregulated in PE placentas. Proteins were extracted from two control and two PE placentas and analyzed. The western blots were probed sequentially with antibodies recognizing the indicated proteins. Two different antibodies were used for MUC1. The antibodies and the molecular weights of each MUC1 protein isoform are indicated at the left and right, respectively

MUC1 has been ascribed activating as well as repressive activities in hypoxia signaling.^43, 44^ Consistent with reports describing MUC1 as an activator of HIF, we found that siRNA-mediated knockdown of *MUC1* mRNA resulted in reduced HIF2A (EPAS1) protein levels, but not *vice versa*, placing MUC1 activity upstream of HIF2A (Figure 5a and Supplementary Figures 4A and B). HIF2A is a transcription factor that induces target-gene expression in response to low oxygen concentration.^40, 45^ Thus, MUC1 positively affects HIF2A-mediated hypoxia responses in BeWo cells. Intriguingly, miR210 is a well-known target of HIF2A^40, 42^ and, thus, would be expected to be responsive to MUC1 regulation. Indeed, we observed decreased miR210 levels not only after knockdown of HIF2A but also upon knockdown of MUC1 (Figure 5b). Because MUC1 is repressed by miR455-3P, miR210 levels are thus kept in check indirectly by miR455-3P (Figure 5c).

The above results are consistent with the miR210 and miR455 levels that we found to be negatively correlated in placenta samples from PE and control patients (Figures 2e and f) and suggest that MUC1 and HIF2A levels are higher in PE than in control samples. As reported previously, we found that HIF2A is expressed in placenta but to markedly higher levels in PE than in control samples (Figure 5d). Importantly, we also detected higher MUC1 protein levels in the placenta of PE patients. Consistent with miRNA-mediated repression of *MUC1* mRNA, different MUC1 protein isoforms increased to the same extent (Figure 5d). In conclusion, PE patients display activated HIF2A-mediated hypoxia signaling in placenta, which may be caused by deregulated expression of miR455-3P.

## Discussion

PE is a severe pregnancy-related disorder in 2–5% of pregnancies in the Occident but complicates up to 10% of pregnancies in developing countries, where emergency care is often inadequate or lacking. Consequently, PE is a leading cause of maternal and fetal/neonatal mortality and morbidity worldwide. Early identification of patients with an increased risk of PE is thus one of the most important goals in obstetrics. This study demonstrates that the use of cellular models can greatly contribute to achieving this goal. On the basis of our preliminary work in a trophoblast tissue culture cell line, we discovered altered expression of three human miRNAs and two proteins in placenta from PE patients. Mechanistic studies of these factors hint at potentially misregulated hypoxia signaling that might contribute to the pathogenesis of PE. Below we discuss the significance of our findings for placental physiology and for the development of tests to diagnose at-risk pregnancies.

Previous studies aimed at the identification of irregular expression of miRNAs in placenta from PE patients revealed increased levels of miR210.^26, 27, 28, 29, 30, 36, 37^ Our results are consistent with these findings and thus validate miR210 as a robust biomarker for PE. Besides miR210, we have identified miR455 as a further prognostic miRNA. Importantly, in contrast to elevated miR210 levels, miR455-3P and miR455-5P levels were significantly lower in PE placenta than in controls. Such a negative correlation could be advantageous for the development of diagnostic assays. Prospective tests assessing miR210/miR455-3P and miR210/miR455-5P ratios may predict PE with high specificity (Supplementary Figure 5). The use of miRNAs as diagnostic markers is interesting for two reasons. First, highly sensitive RT-PCR-based assays can be developed. Second, cell-free nucleic acids, including miRNAs, have been found in maternal plasma.^46^ Notably, the presence of miR455-3P has been suggested in circulating blood.^47^ Thus, measurement of miR210/miR455 ratios in maternal plasma may offer a noninvasive, highly specific and sensitive test of PE risk in early pregnancy.

Unlike most other miRNAs, the pre-miR455 hairpin produces two mature miRNAs, miR455-3P and miR455-5P. We demonstrate that both miR455 miRNAs are part of functional miRISC in BeWo cells, that they are relatively abundant in placenta, and that their expression increases during *in vitro* trophoblast syncytialization. Thus, the two miR455 miRNAs are likely to be implicated in regulatory circuits that are important for placenta development and physiology.

Little is known about the possible regulatory activities of miR455.^48, 49, 50, 51^ Whereas physiological target mRNAs of miR455-5P remain unknown, we have identified *MUC1* mRNA as a *bona fide* miR455-3P target. Corroborating our results, it has been shown recently that MUC1 is also regulated by miR455-3P in lung cells.^52^ Interestingly, both pulmonary and trophoblast cells are exposed to changing oxygen environments and thus mechanisms that buffer against fluctuations in oxygen tension might exist.^53^ Our finding that miR455-3P causes a decrease in HIF2A protein levels indirectly by repressing *MUC1* mRNA (Figure 5a) strongly suggests that miR455-3P may contribute to such buffering.

Insufficient syncytialization of villus CT cells results in suboptimal placental perfusion and thus chronic hypoxia, which is a characteristic of PE. In the first 10 weeks of gestation, the conceptus is in a relatively hypoxic atmosphere^54, 55^ and constraining the activation of a hypoxia response that might counteract syncytialization would be of vital importance. Thus, it is possible that miR455-3P acts as a rheostat restraining a hypoxia response that could otherwise prevent CT to SCT differentiation. Importantly, the reduced miR455 expression in PE samples is unlikely to be simply a consequence of low oxygen tension, because cultivation of BeWo cells under hypoxic conditions did not cause a significant change in miR455 expression (data not shown). Therefore, miR455 expression *per se* may already be irregular, early in the pregnancy of PE patients and thus contribute to pathogenesis. In this respect, it will be very interesting to further investigate regulation of *COL27A1*, the collagen protein-coding gene that hosts the miR455 gene.

In conclusion, although the idea that reduced expression of miR455 is causally linked to the development of PE is intriguing, further experimental evidence to support this model is awaited. Nonetheless, we believe that efforts to develop diagnostics using miRNAs as biomarkers to predict PE should be pursued, irrespective of further mechanistic insight into miR455 function.

## Materials and Methods

### Cell culture and patient recruitment

BeWo cells (ACC 458, DSMZ, Braunschweig, Germany) were grown at 37 °C in a humidified incubator with 5% CO_2_ in Ham's F12 medium supplemented with 20% heat-inactivated fetal bovine serum, penicillin, streptomycin, and glutamine (Life Technologies, Carlsbad, CA, USA). Forskolin (FSK, 344270) was purchased from Merck Millipore (Billerica, MA, USA) and DMSO from Sigma-Aldrich (St. Louis, MO, USA) (D2650).

The prospective case–control study was approved by the local ethical committee. After written informed consent, placenta was collected and processed within 15 min of the delivery. All patients underwent either elective cesarean section (CS, controls, *n*=14) or scheduled CS due to severe PE (*n*=15). Severe PE was defined as a blood pressure of ≥160/100 mmHg confirmed after an interval of at least 6 h, in combination with a proteinuria of ≥2+ (dipstick) recorded at least twice within 24 h. Patient data are summarized in Table 1.

### 3′UTR cloning and dual luciferase assay

To construct the UTR vectors, a psicheck-2 vector (Promega, Madison, WI, USA) containing an Asc1 site was created. Briefly, the psicheck-2 vector was first digested with *Not*1/*Xho*1, purified on a 1% agarose gel and extracted using QIAquick Gel Extraction Kit (Qiagen, Venlo, The Netherlands). The linearized vector was ligated to annealed oligonucleotides containing an Asc1 restriction site (Asc1 fwd and Asc1 rev, Supplementary Table S2). The vector was digested using Asc1/Not1 enzymes and ciped (except for vectors containing perfect complementary sequences for miR455-3P and -5P).

3′UTRs were amplified from total RNA extracted from BeWo cells. Briefly, total RNA was reverse transcribed following a first-strand cDNA synthesis protocol from an AffinityScript Multiple Temperature cDNA synthesis kit (Agilent, Santa Clara, CA, USA) and amplified using an iProof High-Fidelity PCR kit (Biorad, Hercules, CA, USA). Oligonucleotides were designed to amplify specifically the different UTRs using the NCBI reference gene and UCSC genome browser (Supplementary Table S2) (except for the longest HIF1AN 3′UTR, which is not amplified/found in BeWo cells; we amplified the shortest UTR from the Ensembl genome browser). The amplified UTRs were digested using *Mlu*1/*Not*1 enzymes.

Digested vector and amplified 3′UTRs were ligated using a Rapid DNA ligation kit (Roche Diagnostics, Basel, Switzerland). For control vectors, oligonucleotides containing perfect complementary sequences for miR455-3P or -5P (Supplementary Table S2) were annealed and ligated to unciped digested vector.

BeWo cells were transiently transfected with luciferase reporter constructs following a Nanofectin protocol (PAA). At 48 h posttransfection, cells were lysed and luciferase activity measured using the Dual Luciferase Reporter assay system (Promega). RL activities were normalized to FL activity . Measurements were carried out in technical triplicates and are the results of three independent biological experiments. Data are presented either as RL/FL ratios or as percentage repression (ratio RL/FL in FSK conditions normalized to the ratio in DMSO conditions).

### Transient transfection siRNA and mimics

siRNA (*MUC1*, *HIF2A*, All Stars Negative Control) and synthetic miRNA/mimics (hsa-miR455-3 P and -5P) were purchased from Qiagen. Transient siRNA and miRNA mimic transfections in BeWo cells were performed with RNAimax (LifeTechnologies) following the manufacturer's protocol.

### RNA isolation and expression analysis

Total RNA with or without miRNAs was extracted from BeWo cells and placenta pieces using an mirVana miRNA Isolation Kit (LifeTechnologies). The RNA used for pri-miRNA 455 quantification was treated further with a Turbo DNA-free Kit following the recommendations of the supplier (Life Technologies).

For the placenta, small pieces (<150 mg) were dissected from the villus tree within 15 min of the delivery. After extensive washing in cold PBS, samples were stored for 24 h at 4 °C in an RNAlater solution (Life Technologies), dried, and stored at −80 °C. Frozen tissue was directly transferred to pre-chilled lysis solution, homogenized using a Polytron PT 2100 (Kinematica AG, Luzern, Switzerland), and then processed as for the cells. The quality of placental RNA samples was estimated using total RNA Chip on an Agilent 2100 Bioanalyzer. Only samples with a RIN value >7.5 were considered for further experiments.

For mRNA quantification, quantitative qRT-PCR was performed with a TaqMan One Step RT-PCR Master Mix reagents kit (LifeTechnologies). To evaluate miRNA and pri-miRNA expression, RT- PCR was performed using a TaqMan MicroRNA reverse transcription kit and a High Capacity RNA to cDNA kit, respectively, followed by a TaqMan Universal Master Mix, no UNG (Life Technologies).

The primers used for qPCR experiments were purchased from Life Technologies and are available upon request. All experiments were performed in triplicate using the StepOne plus real-time PCR system for 96-well plates or the 7900HT Fast real-time PCR system for 384-well plates (Life Technologies). All mRNA and miRNA data were normalized to RPLP0 and U6snRNA, respectively, except when stated otherwise.

### Preparation of small RNA libraries for high-throughput sequencing and bioinformatic analysis

The protocol from Emmerth *et al.*^56^ was adapted for human small RNA libraries. After total RNA extraction from BeWo cells using a miRVana kit (Life Technologies), 17- to 30-nt small RNAs were PAGE-purified and cloned based upon the preactivated, adenylated linkering method described previously^57^ using a mutant T4 RNA ligase (Rnl2 1-249).^58^ All samples were barcoded at the 3′end of the 5′ adapter using a hamming distance two code with a 3′cytosine (AAAC, ACCC, AGGC, ATTC, CACC, CCGC, CGTC, CTAC, GAGC, GCTC, GGAC, GTCC, TATC, TCAC, TGCC, TTGC) and sequenced in one lane of an Illumina GAIIx instrument (Illumina Inc., San Diego, CA, USA).

Individual reads were assigned based on the first four nt containing the barcode. The 3′adaptor was removed by aligning it to the read, allowing one or two mismatches in prefix alignments of at least seven or ten bases, respectively. Low-complexity reads (<1%) were filtered out based on their dinucleotide entropy. All reads shorter than 14 nt were removed. Alignments to the *Homo sapiens* miRNA database (Human Genome Assembly hg19, mirBase v15) were performed with the software Bowtie (version 0.12.7, http://bowtie-bio.sourceforge.net).^59^ The numbers of miRNA reads were normalized to the total number of reads of the library. For the purpose of logarithmic scale representation, a number of 2 reads was added to each normalized miRNA number. All statistical and bioinformatic analyses were carried out in R using Bioconductor.

### Protein isolation and western blotting

Total cellular protein was extracted using RIPA buffer (50 mM Tris-HCl pH 7.5, 150 mM NaCl, 1% NP-40, 0.5% sodium deoxycholate, 0.1% SDS, 1 mM EDTA) supplemented with the Protease and Phosphatase Inhibitor Cocktail (Roche Applied Science). For the placenta, pieces were dissected as described for RNA preparation but were snap frozen in liquid nitrogen and stored at −80 °C. Frozen tissues were thawed for a few minutes in pre-chilled RIPA buffer (1 ml per100 mg), homogenized with a Polytron (Kinematica), and sonicated twice for 1 min (pulsed 2 s on/2 s off). After centrifugation (13 000 rpm., 15 min), 20–40 *μ*g aliquots of protein were separated on MiniProtean TGX precast gels (Biorad) and transferred to a nitrocellulose membrane (Protran, Whatman). The membrane was blocked for 1 h at room temperature(RT) in PBS containing 0.05% tween-20 and 5% non-fat dry milk, incubated overnight at 4 °C with primary antibodies, and for 1 h at RT with an HRP-conjugated secondary antibody. Fractions were detected by Western Lightning Plus ECL (Perkin Elmer, Waltham, MA, USA). This study used primary antibodies against EPAS1/HIF2A (NB 100-122), FIH1/HIF1AN (EPR3658, NBP1-40688), TBP (NB 500-700), and MUC1 (EP1024Y, NB110-57234) from Novus Biologicals (Littleton, CO, USA). ARNT (ab2771), EGLN2 (ab108980), and MUC1 (ab101352) were purchased from Abcam (Cambridge, MA, USA).

### Immunofluorescence in BeWo cells

Coverslips were cleaned by ethanol:chloric acid (99 : 1) wash. One coverslip was deposited into each well of a six-well plate and sterilized by UV treatment. BeWo cells were plated at a density of 50 000 cells per well. Cells after settling for 1 day were treated with DMSO or FSK for 48 h, washed in PBS twice at RT, and fixed in 2% PBS-PFA for 5 min at RT. The PFA was blocked by adding 0.125 M glycine for 5 min. After extensive PBS washing, cells were permeabilized with 0.1% PBS-Triton X-100 for 5 min. The slides were transferred to a humid chamber and, after 30 min blocking in PBS-BSA (10%) were incubated with primary antibody against CDH1 (ab1416, dilution 1/50, Abcam) in PBS-BSA (1%) overnight at 4 °C. Cells were washed and incubated with secondary antibody (A-11029, dilution 1/1000, Life Technologies) in PBS-BSA (1%) for 1 h at RT. After washing, DAPI was applied at a dilution of 1/10000. After extensive washing, the coverslips were mounted on slides with MOWIOL mounting medium. Fluorescent images were acquired using a Zeiss Axioplan 2 Imaging inverted fluorescence microscope in conjunction with a Zeiss Axiocam MRm monochromatic CCD camera or Zeiss Axiocam MRc color CCD camera and analyzed with Axiovision 4.8.2 software (Carl Zeiss AG, Oberkochen, Germany).

## Figures and Tables

**Table 1:**
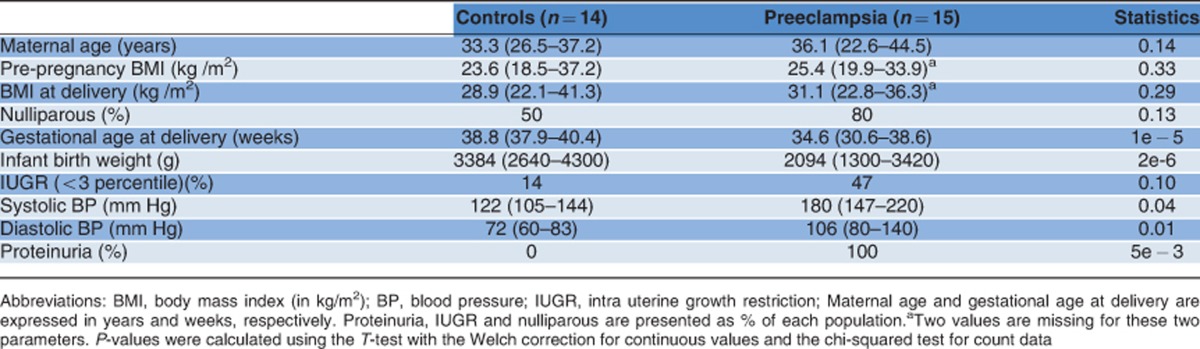
Clinical parameters of the control and Preeclampsia women recruited for the study

## Abbreviations

miRNA: microRNA
PE: preeclampsia
nt: nucleotide
pri-miRNA: primary microRNA
pre-miRNA: precursor microRNA
miRISC: microRNA induced silencing complex
UTR: untranslated region
C14MC: chromosome 14 microRNA cluster
C19MC: chromosome 19 microRNA cluster
CT: cytotrophoblast
SCT: syncytiotrophoblast
FSK: forskolin
DMSO: dimethylsulfoxid
DAPI: 4′,6′-diamidino-2-phénylindole
CGB: beta chorionic gonadotropin hormone
snRNA: small nuclear RNA
RL: renilla luciferase
FL: firefly luciferase
h: hours
HIF: hypoxia inducible factor
TBP: TATA binding protein
Ctrl: control
Ct: cycle threshold
RT-PCR: reverse transcription-polymerase chain reaction

## Notes

A patent application has been filed.
