# Supplementary material for: miR455 is linked to hypoxia signaling and is deregulated in preeclampsia
Source: Cell Death Dis. 2014 Sep 4;5(9):e1408–. doi: 10.1038/cddis.2014.368 (PMC4540200; doi:10.1038/cddis.2014.368)
Supplement: Supplementary Data [file cddis2014368x3.doc]

**Supplementary Data**

**miR455 is linked to hypoxia signaling and is deregulated in preeclampsia**

Sébastien Lalevée, Olav Lapaire, and Marc Bühler

**Supplementary Figures**

**
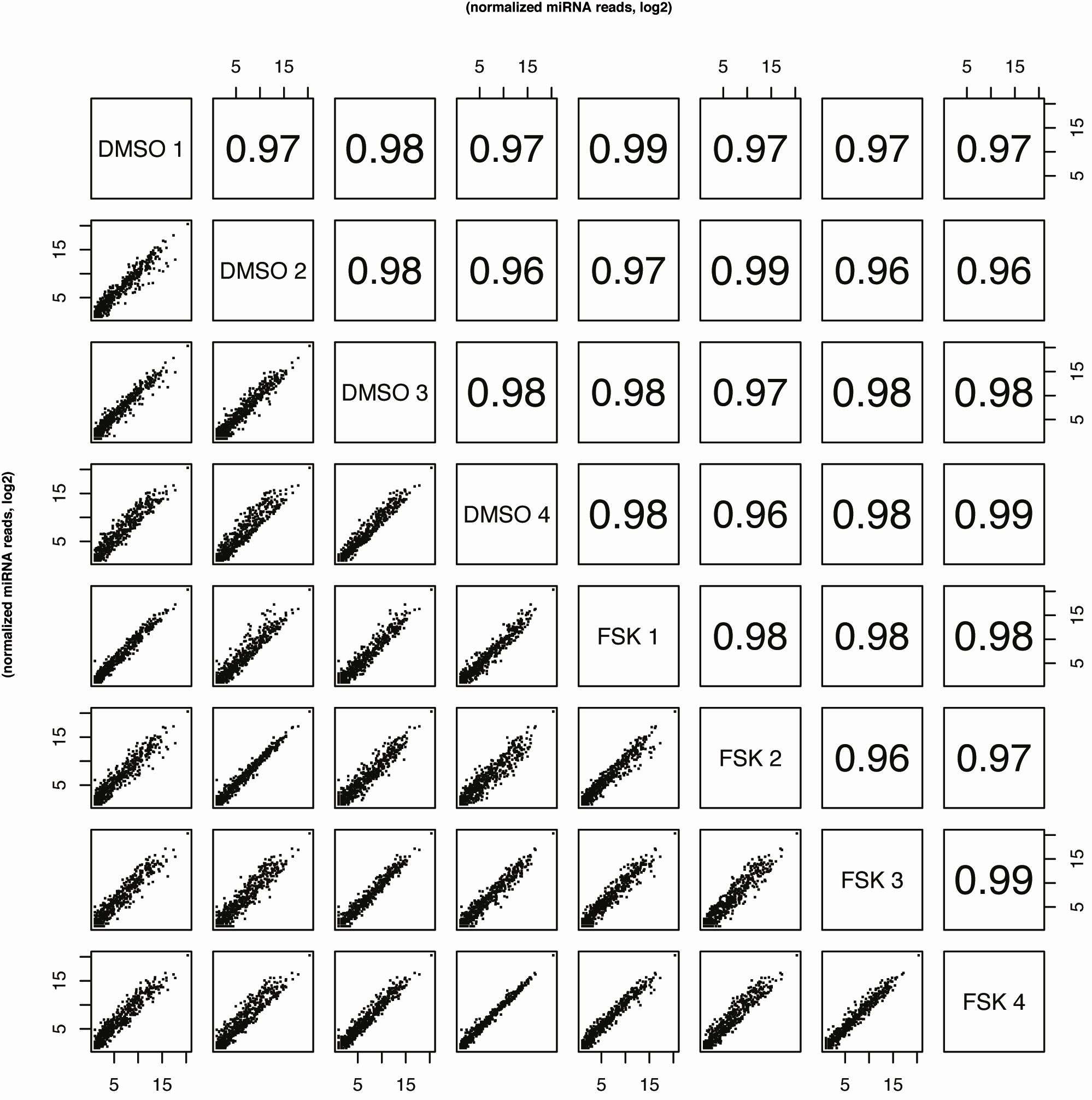
**

**Supplementary Figure 1.** Pairwise comparison of miRNA expression profiles obtained by small RNA sequencing. Four biological replicates were performed for each DMSO and FSK treatment (1-4, respectively). Pearson’s correlation coefficients are indicated.

**
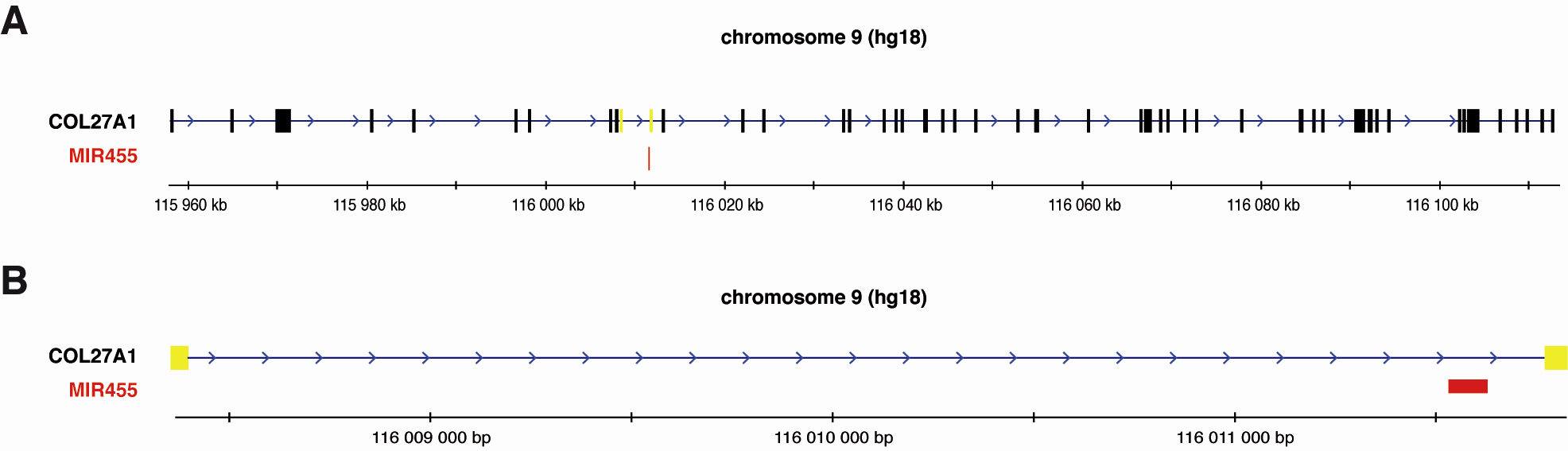
**

**Supplementary Figure 2. A.** Schematic view of the human Col27A1 gene. Exons and introns are depicted as black boxes and a blue line, respectively. miR455 and its two neighboring exons are represented as one red and two yellow boxes, respectively. **B.** Schematic view of intron 10 of the Col27a1 gene. miR455 is indicated in red.

**
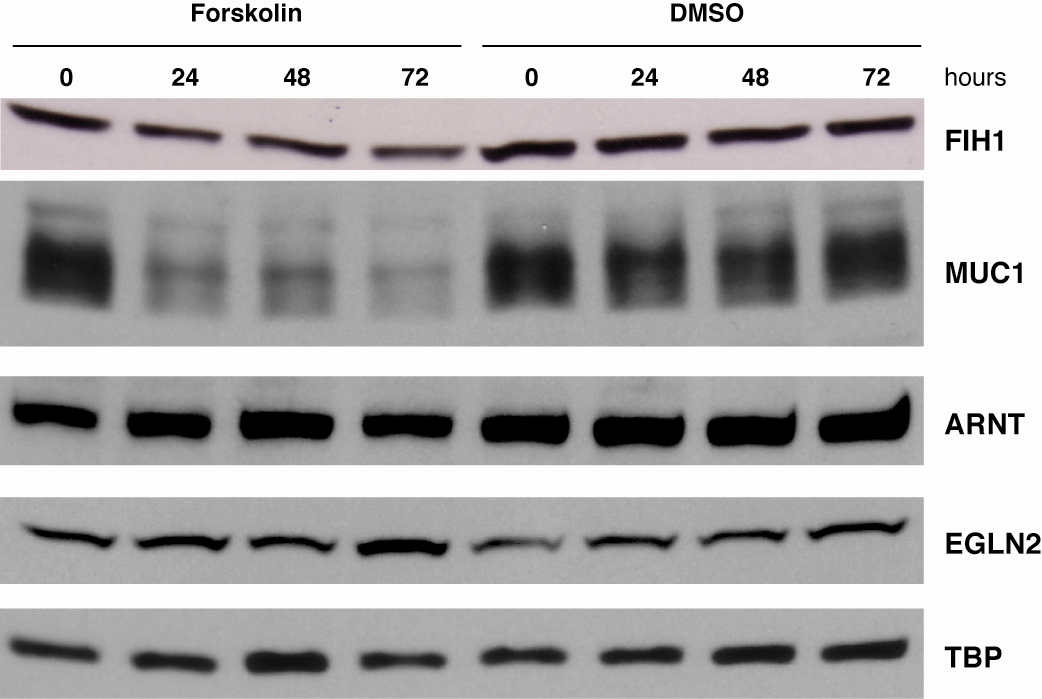
**

**Supplementary Figure 3.** Protein levels of potential miR455 target genes (MUC1, FIH1, ARNT and EGLN2). BeWo cells were treated with DMSO or FSK and harvested every 24 h. Protein samples were analyzed by western blotting. TBP served as a loading control.

**
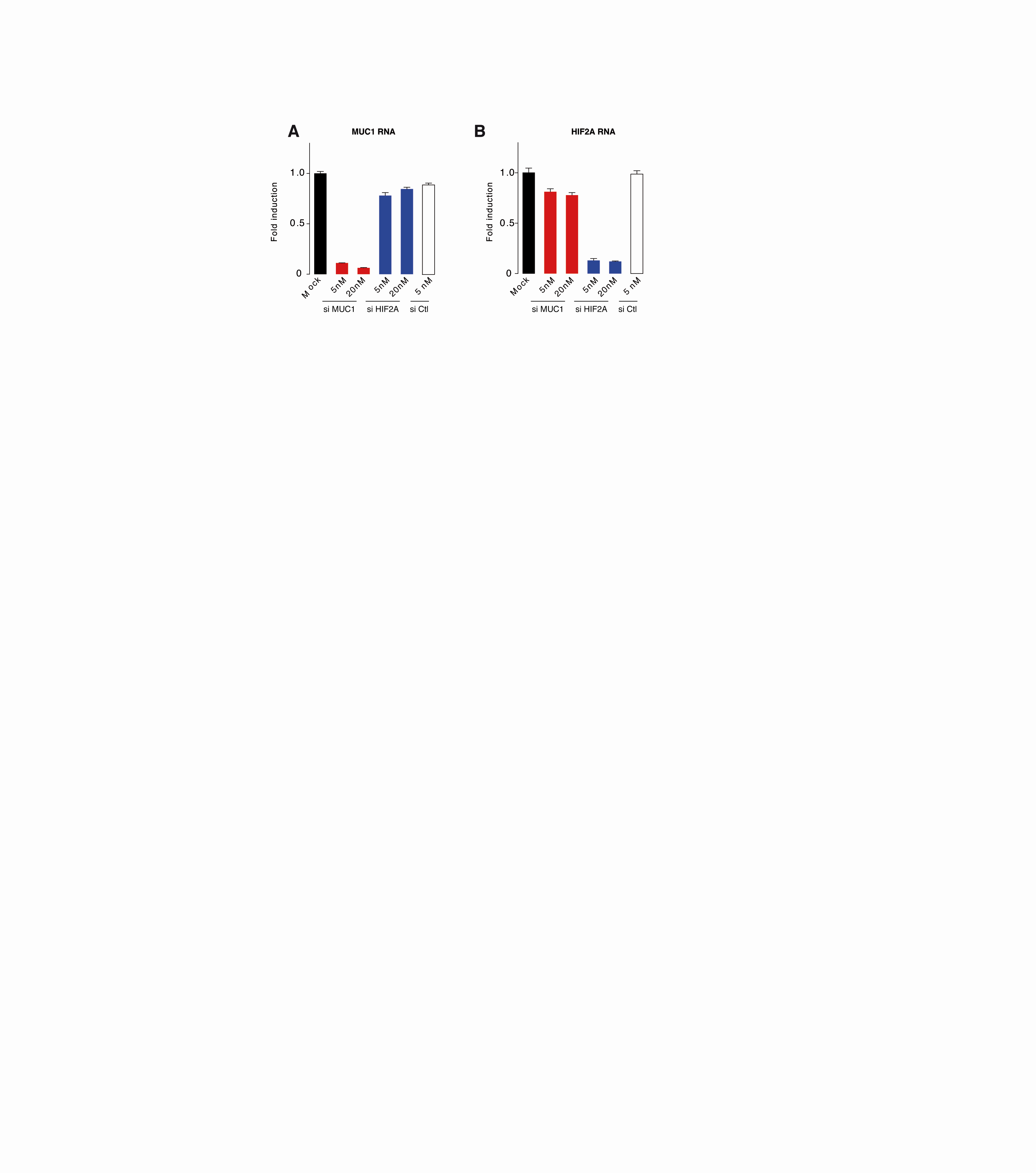
**

**Supplementary Figure 4A, B**. BeWo cells were transfected with siRNA against MUC1 or HIF2A (each at concentrations of 5 and 20 nM) or control siRNA at 5 nM (All Stars Negative Control). RNA was isolated 48 h post-transfection. Expression of MUC1 (A) or HIF2A (B) was analyzed by qRT-PCR and normalized to RPLP0 mRNA levels (median value +/- SEM of three independent experiments).

**
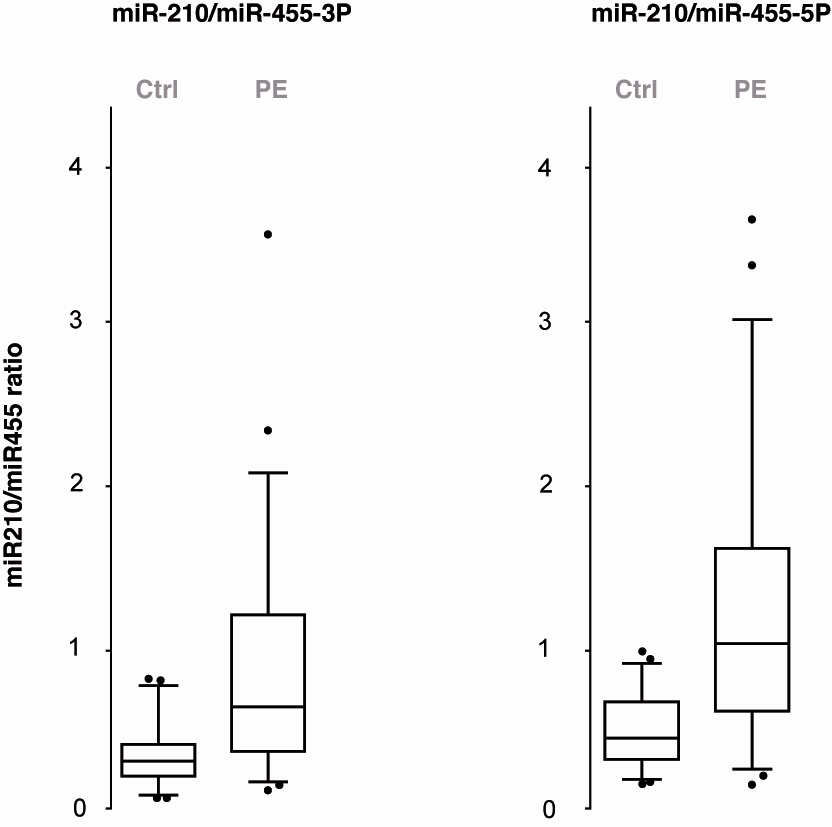
**

**Supplementary Figure 5.** The miR210/miR455 ratio may serve as a predictive value to diagnose preeclampsia. miRNAs were detected by quantitative real-time RT-PCR using miRNA-specific TaqMan assays. For each placenta, miR210 Ct values were normalized to miR455-3P (*left*) or miR-455-5P (*right*) Ct values. Ratios were calculated using the DDCt method and plotted using Whiskers Box Plot 1-99 percentile representation.
